# Interaction of saffron and its constituents with Nrf2 signaling pathway: A review

**DOI:** 10.22038/IJBMS.2022.61986.13719

**Published:** 2022-07

**Authors:** Arian Khoshandam, Bibi Marjan Razavi, Hossein Hosseinzadeh

**Affiliations:** 1 School of Pharmacy, Mashhad University of Medical Sciences, Mashhad, Iran; 2 Targeted Drug Delivery Research Center, Pharmaceutical Technology Institute, Mashhad University of Medical Sciences, Mashhad, Iran; 3 Department of Pharmacodynamics and Toxicology, School of Pharmacy, Mashhad University of Medical Sciences, Mashhad, Iran; 4 Pharmaceutical Research Center, Pharmaceutical Technology Institute, Mashhad University of Medical Sciences, Mashhad, Iran

**Keywords:** Crocetin, Crocin, Crocus sativus, Nrf2, Safranal, Saffron

## Abstract

Saffron (*Crocus sativus*) is a natural compound and its constituents such as crocin, crocetin, and safranal have many pharmacological properties such as anti-oxidant, anti-inflammatory, antitumor, antigenotoxic, anti-depressant, hepatoprotective, cardioprotective, and neuroprotective. The nuclear factor erythroid 2-related factor 2 (Nrf2) signaling pathway plays an important role against inflammation, oxidative stress, and carcinogenesis. In the regulation of the Nrf2 signaling pathway, kelch-like ECH-associated protein 1 (keap1) is the most studied pathway. In this review, we gathered various studies and describe the pharmacological effects of saffron and its constituents with their related mechanisms of action, particularly the Nrf2 signaling pathway. In this review, we used search engines or electronic databases including Scopus, Web of Science, and Pubmed, without time limitation. The search keywords contained saffron, “*Crocus sativus*”, crocetin, crocin, safranal, picrocrocin, “nuclear factor erythroid 2-related factor 2“, and Nrf2. Saffron and its constituents could have protective properties through various mechanisms particularly the Nrf2/HO-1/Keap1 signaling pathway in different tissues such as the liver, heart, brain, pancreas, lung, joints, colon, etc. The vast majority of studies discussed in this review indicate that saffron and its constituents could induce the Nrf2 signaling pathway leading to its anti-oxidant and therapeutic effects.

## Introduction

**Figure 1:**
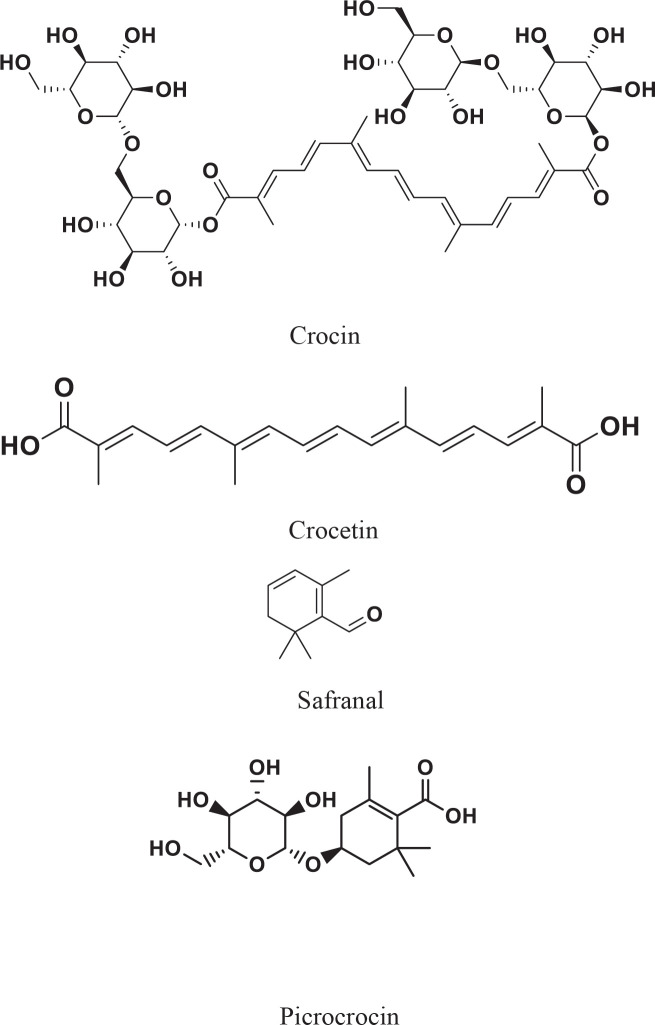
Chemical structures of saffron bioactive constituents

*Crocus sativus* is a plant that belongs to the Iridaceae family (1). Saffron is derived from the stigmas of *C. sativus* (2, 3). It is known as “red gold” and is widely used and highly priced due to its arduous handling and processing method (4). It mainly contains specialized metabolites including picrocrocin, crocins, safranal, as well as crocetin (Figure 1) (6). The odor and aroma of saffron are due to safranal (7). Crocin and crocetin are responsible for the color of saffron and have revealed many pharmacological properties such as anti-oxidant, antidepressant, neuroprotective, and vasodilation (1, 8). Crocins are a series derived from crocetin glycosides (9) and have many health benefits such as anti-oxidant (10), neuroprotectant (11), anticancer (12), antidepressant (13, 14), hepatoprotective (15), cardioprotective (16), and antihyperlipidemic (17). Several important properties of saffron have been correlated to crocetin and it has mechanisms of action such as elevation of the rate of oxygen transport, induction of apoptosis in cancer cells, and cell protection from reactive oxygen species (ROS). Thus, crocetin can be effective in hemorrhages, tumors, atherosclerosis (18, 19), etc. Saffron and its components are pharmacologically effective in various parts of the body such as the cardiovascular system (20, 21), immune system (22), nervous system (8, 23), genital system (24) and eye (14). In addition, some other effects of saffron are anti-tumor (25), anti-inflammatory (26), anti-oxidant (27), antidotal (28), antigenotoxic (29), and chemoprotective properties (29).

A transcription factor named Nuclear factor erythroid 2-related factor 2 (Nrf2) has an important role in keeping the balance between anti-oxidant and oxidant systems by activating heme oxygenase-1 (HO-1) (30). The other name of Nrf2 is NFE2L2 which has a cap “n” Collar basic leucine zipper structure (31, 32). Bach1, Bach2, Nrf1, Nrf2, Nrf3, and NF-F2 are 6 members of this family in mammals that have their characteristic functions. Three members of this family that have been investigated more include Nrf1, Nrf2, and Nrf3. Nrf1 is mainly responsible for expression of the proteasome gene (33). Nrf3 plays a vital role in inflammation, carcinogenesis, and differentiation (35). Genes containing anti-oxidant response elements (ARE) are bound to Nrf2. Thus, Nrf2 participates in pivotal molecular processes such as metabolism of iron and heme, repairment of DNA, proteostasis, and regulation of redox (36). Responding to the exogenous and endogenous tensions by altering the defense mechanisms is the most important role of the Nrf2 signaling pathway (37). Up to now, various studies have been done to realize downstream targets and biological activities of the Nrf2 signaling pathway and also its potential role as a target in the treatment of diseases, particularly cancer (38–44). Under oxidative stress and the consequent possible damage, the organs such as the brain, liver, and kidney increase the expression of Nrf2 (45). Kelch-like ECH-associated protein 1 (keap1), epigenetics, and PI3K/Akt are three pivotal pathways that regulate Nrf2 signaling (46). The most important and studied pathway in the regulation of the Nrf2 signaling pathway is keap1. keap1 induces Nrf2 ubiquitination via complex known cullin 3-based E3 ligase in physiological conditions that causes its decomposition by 26S proteasome and consequently in this mentioned process, translocation of Nrf2 from the cytoplasm to the nucleus is inhibited. Thus, due to this mechanism, the level of Nrf2 in the cytoplasm is preserved at the least rate (47). Oxidative stress has damaging effects on the cells and the Nrf2 signaling pathway has the most important role in this condition via its redox ability. Because of the modification of cysteine residue of keap1, the interaction between keap1 and Nrf2 does not happen. Hence, the complex of Nrf2-keap1 is disrupted and Nrf2 accumulates in the cytoplasm with high concentrations leading to its translocation to the nucleus and triggering several cytoprotective events such as molecular chaperones, anti-oxidant and phase II detoxification enzymes, and anti-inflammatory (48, 49). 

**Figure 2:**
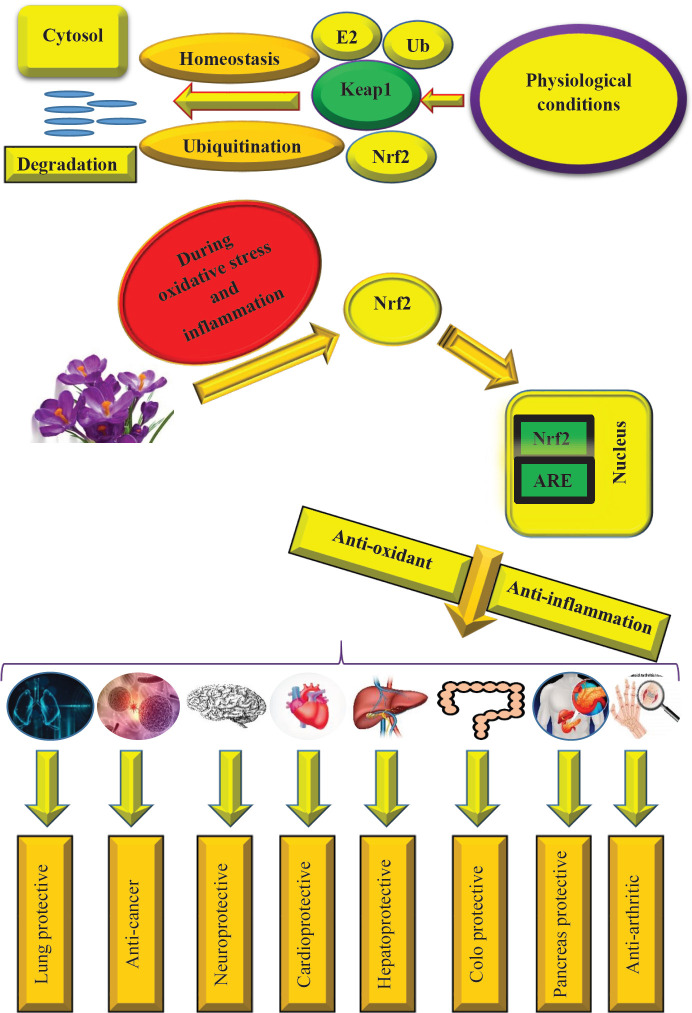
Effects of saffron on the Nrf2 signaling pathway

In this review, we gathered various studies about the biological and therapeutic activities of saffron and its constituents such as crocin, crocetin, and safranal as well as their mechanisms particularly the Nrf2 signaling pathway (Table1) (Figure 2). 

**Search method**

In this review, we used search engines or electronic databases including Scopus, Web of Science, and Pubmed, without time limitation. The search keywords contained saffron, “*Crocus sativus*”, crocetin, crocin, safranal, picrocrocin, “nuclear factor erythroid 2-related factor 2“, and Nrf2. 

**Effects of saffron and its constituents on the Nrf2 signaling pathway**

***Hepatoprotective effects***

 Akbari *et al*. (2016) evaluated the effects of seven days of crocin (200 mg/kg) treatment on liver ischemia-reperfusion (IR) injury in male Wistar rats. The expression of Nrf2 was also increased in the crocin treatment group compared with the IR group, consequently, ROS was reduced. Crocin enhanced the decreased level of anti-oxidant capacity by increasing the level of superoxide dismutase (SOD), CAT, and glutathione peroxidase (GPx). Therefore, crocin protected the liver of rats from IR-induced injury and it showed hepatoprotective properties (50).

In another study, the anti-oxidant and hepatoprotective effects of crocin (25 or 50 mg/kg daily for 7 days.) in arsenic trioxide (ATO)-induced hepatic injury in rats have been investigated. Crocin decreased oxidative stress and ROS via increasing Nrf2, HO-1, nicotinamide adenine dinucleotide quinone dehydrogenase 1 (NQO1) in ATO-induced hepatic injury. Hence, crocin could be a natural and safe agent to prevent hepatotoxicity in ATO-induced hepatic oxidant stress and it could be a therapeutic option in patients with acute promyelocytic leukemia (51).

The protective effects of crocin (60 mg/kg) on liver function in Sprague-Dawley rats with traumatic hemorrhagic shock (THS) have been studied. Crocin significantly enhanced tissue blood flow, rate of survival, hemodynamic parameters, and improved liver function in THS rats. The underlying mechanism of the hepatoprotective effect of crocin is its ability to reduce oxidative stress and ROS through enhancement of gene expression of Nrf2, HO-1, and anti-oxidant enzymes, such as CAT, GSH, and SOD (52).

In an experiment on rat liver cell line (BRL-3A), the effects of total flavonoid extract of saffron petals (TFESP), stamens (TFESS), and the mixture of stamens and petals (TFEMS) on oxidative stress and liver injury have been evaluated. Tert-butyl hydroperoxide (t-BHP) was used to induce oxidative stress and liver injury in cells. ROS causes many pathological conditions, such as inflammation, liver injury, and apoptosis. TFESP showed a stronger scavenging effect than TFESS and TFEMS because its flavonoid content demonstrated a higher activity than TFESS and TFEMS. TFESP (300 μg/ml for 1 day) showed no cytotoxicity and it was able to return t-BHP-induced hepatic damage due to its ability to reduce ROS accumulation in liver cells induced by t-BHP and elevate the expression of genes related to anti-oxidant pathways including keap1, Nrf2, and HO-1 in hepatocytes. TFESP could also increase the enzymes with anti-oxidant activity such as catalase (CAT), and glutathione (GSH) (53).

Cumulatively, saffron and its constituents could have hepatoprotective properties through the suggested mechanisms such as inducing the Keap1/Nrf2/HO-1 signaling pathway leading to anti-oxidant properties.

***Cardioprotective effects***

The cardioprotective effect of crocin (40 mg/kg/day and 80 mg/kg/day for 7 days) in ATO-induced cardiac injury in male adult Sprague-Dawley rats has been studied. Crocin ameliorated cardiotoxicity in ATO-induced rats through mechanisms such as Nrf2 activation leading to increased keap1, HO-1, and NQO1 involved in the Nrf2 signaling pathway and decreasing ROS and oxidative stress. Nuclear factor kappa-light-chain-enhancer of activated B cells (NF-κB) is the potential mechanism for oxidative stress. Under ATO exposure, ROS excessive production leads to NF-κB activation. A pivotal inducer of NF-κB is tumor necrosis factor-alpha (TNF-α) exposure, which caused its enhancement. There is an interaction between the Nrf2 signaling pathway and NF-κB. Activating of Nrf2 signaling pathway via crocin treatment reduced NF-κB, inflammation, and oxidative stress. Oxidative stress induced by ATO treatment caused cardiac apoptosis. B cell lymphoma-2 (Bcl-2) protein family is an anti-apoptotic member. In this study, Nrf2 up-regulated Bcl-2. Crocin also enhanced the decreased level of anti-oxidant enzymes including SOD, CAT, and GPx in the heart of ATO-induced rats. Therefore, crocin could have beneficial impacts on cardiac health (54).

R. León *et al*. (2017) in both *in vivo* and *in vitro* experiments, evaluated the cardioprotective effects of standardized saffron aqueous extract (SFE) (60 µg/kg bolus 15 min and 60 mg/kg/day for 4 weeks) in IR-induced injury in Wild-Type (WT) and ApoE^(_/_) ^mice. For the *in vitro* part of the study, the AREc32 cell line has been evaluated. The results of this experiment revealed that SFE in both WT and ApoE^(_/_)^ mice with myocardial injury induced cardioprotection. Besides saffron administration demonstrated a beneficial impact on endothelial dysfunction. This study showed that SFE can induce Nrf2 in both *in vivo* and *in vitro* conditions leading to enhancement of HO-1, NQO1, MnSOD, GSH, and nicotinamide adenine dinucleotide phosphate (NADPH) and decreasing ROS and oxidative stress. Besides, the levels of malondialdehyde (MDA) and 3-nitrotyrosine (NT) were diminished by SFE which is related to its anti-oxidant and anti-inflammatory properties. SFE treatment increased protein kinase B (Akt) and extracellular signal-regulated kinases (ERK1/2). Akt and ERK1/2 can activate the Nrf2 signaling pathway. Akt activation causes glycogen synthase kinase 3 beta (GSK3b) deactivation and decreases signaling for proteasomal degradation of Nrf2. Hence, SFE could increase the Nrf2 signaling pathway in these parallel pathways and could have beneficial effects on cardiac health (55).

In another experiment, the effects of crocetin (10 μM, 20 μM, and 40 μM) against IR injury in an *ex vivo* experiment in Sprague-Dawley rats have been demonstrated. This study revealed that unfolded protein response (UPR) increased in IR injury conditions in rats. Crocetin diminished inflammation via up-regulating the Nrf2 signaling pathway which led to NQO1, HO-1, and NADPH enhancement and also down-regulated UPR. Nrf2-deficient cells demonstrated a lower crocetin cardioprotective effect which could be evidence that the cardiac protective properties of crocetin are Nrf2 dependent but the relation between Nrf2 and UPR remains unclear. Thus, crocetin could be a therapeutic option in cardiac diseases due to its anti-oxidant and anti-inflammatory effects (56).

Endoplasmic reticulum (ER) stress is correlated with IR-induced heart cell apoptosis. Wang *et al.* (2018) evaluated the cardioprotective properties of crocin (50 mg/kg/day for 7 days) in C57BL/6 mice undergoing ER stress and IR injury. The intensity of ER stress is associated with the level of glucose-regulated protein of 78 kD (GRP78) expression. C/EBP homologous protein (CHOP) is another hallmark of ER stress and can induce apoptosis. The enhanced level of GRP78 and CHOP in IR injury, greatly decreased in both *in vivo* and *in vitro* conditions by crocin. Hence, crocin could ameliorate the IR-induced apoptosis in cardiomyocytes by suppressing ER stress. miR-34a is a miRNA that is correlated with various IR injuries. Crocin treatment significantly decreased the expression of miR-34a. The silent information regulator 1 (Sirt1)/Nrf2 pathway is closely associated with oxidative stress. The expression of HO-1, Sirt1, and Nrf2 signaling pathways was notably increased by crocin treatment. Therefore, crocin could have cardioprotective, anti-apoptotic, and ER suppressing properties by down-regulation of miR-34a and activation of the Sirt1/Nrf2 pathway (57).

The cardioprotective effects of crocetin (50, 100, and 200 mg/kg/day/ for 15 days) in isoproterenol-induced myocardial infarction in Wistar rats have been investigated. During myocardial infarction, the level of lactate dehydrogenase (LDH) and creatine kinase enzymes was enhanced. But crocetin treatment reduced the elevated levels of these enzymes towards normal. The suggested mechanism of the cardioprotective effects of crocetin was its anti-oxidant potential. In the myocardial tissue, oxidative stress increased and induced the apoptosis process via the mitochondrial pathway by BCL2 Associated X (Bax) and caspase enzyme. Administration of crocetin reduced the level of Bax and caspase leading to its apoptosis reduction ability in the myocardial tissues of rats. Additionally, crocetin increased the Nrf2 signaling pathway and decreased MDA and SOD contents. Thus, it could be beneficial for cardiac health due to its anti-inflammatory and anti-oxidant properties (58).

Taken together, saffron and its constituents could have cardioprotective properties by increasing NQO1, Akt, ERK1/2, and decreasing GSK3b resulting in inducing the Nrf2/HO-1 signaling pathway, increasing the gene expression of anti-oxidant enzymes such as SOD, CAT, and GPx, reducing GSK3b, NF-κB expression, MDA, and ROS leading to anti-oxidant and anti-inflammatory properties.

***Chemo-preventive effects***

In an experiment, thioacetamide (TAA) (200 mg/kg, IP) was used to induce hepatocarcinogenesis in Sprague-Dawley rats, and crocin (10 mg/kg, IP daily for 4 weeks) effects on cancer have been demonstrated. This study revealed that HCC progression is closely associated with oxidative stress and suppression of the Nrf2 signaling pathway. Crocin treatment, induced Nrf2, HO-1, keap1, SOD, GPx, GST, and GSH and reduced MDA. Thus, an important reason for the chemoprotective-protective effects of crocin is its anti-inflammatory and anti-oxidant functions (59).

The effects of crocin (0, 50, 100, and 200 ppm for 15 weeks) against colon carcinogenesis and colitis induced by chemicals in male ICR mice have been investigated. Azoxymethane (AOM) and dextran sodium sulfate (DSS) were used to induce colonic adenocarcinoma. The Nrf2 expression was enhanced via crocin treatment leading to its anti-oxidant effects and its possible therapeutic role in the suppression of colitis-related colorectal carcinogenesis and cancer. No histopathological and clinical toxicity was observed under crocin treatment. Proliferation activity was also suppressed by crocin administration. Thus, crocin could be an anti-proliferative and anti-carcinogenesis factor due to its anti-inflammatory and anti-oxidant properties. NF-*κ*B has a vital role in inflammation-associated carcinogenesis. In the colorectal mucosa of mice that had DSS in their diet, NF-*κ*B expression was increased which was inhibited by crocin administration. mRNA expression of genes that are involved in the NF-*κ*B activation including cyclooxygenase- (COX-) 2, inducible nitric oxide synthase (iNOS), TNF-α, and interleukin (IL-) 1*β* were lower in the DSS plus crocin group compared with the DSS given group (60).

In an *in vitro* study on human gastric mucosa epithelial cell line GES-1 induced by 1-methyl-3-nitroso-1-nitroguanidine (MNNG), the effects of crocin (0–100 μM for 6 weeks) on cell cycle, apoptosis process, proliferation, and epithelial-mesenchymal transition (EMT) have been revealed. MNNG was used to induce cancer in GES-1 cells. Crocin, diminished invasion and migration. The level of Nrf2 and downstream gene expression including NQO1 and HO-1 was significantly reduced and tafazzin (TAZ) and yes-associated protein (YAP) levels increased in the malignant cells. Crocin treatment reversed these effects. Two Hippo signaling pathway co-activators are TAZ and YAP. The hippo pathway is associated with development of the organ, metastasis, and cell proliferation. This study has revealed that crocin treatment, enhanced Nrf2 expression to suppress the Hippo pathway, thereby alleviating the progression of cancer in GES-1 cells. Moreover in this study, crocin elevated the Bax/Bcl2 ratio and caspase activation leading to apoptosis in malignant cells (61).

The cytotoxicity of crocetin and crocin in 5 cancer cell lines including adenocarcinoma alveolar basal cell line (A549), hepatocellular liver cell line (HepG2), colorectal colon epithelial cell line (HCT-116), adenocarcinoma cervical epithelial cancer cell line (HeLa), and adenocarcinoma ovarian epithelial cell line (SK-OV-3) has been evaluated. Both crocetin and crocin increased Nrf2 expression and up-regulated NQO1, NQO2, and HO-1 in HeLa cells but the activation effect of crocetin on Nrf2 was higher than crocin. One of the chemopreventive treatments that suppress lactate formation is the inhibition of LDHA. LDHA inhibition also decreases the membrane potential and the levels of ATP in mitochondria, leading to oxidative stress induction in hypoxic cancer cells. In crocetin and crocin-treated HeLa cells, induction of ROS by inhibition of LDHA contributed to activation of the Nrf2 signaling pathway. Crocetin and crocin diminished LDHA expression by 34.2% and 10.5%, respectively in comparison with the control in HeLa cells. Crocetin induced a notable ROS amount in HeLa cells but crocin did not. *N*-acetylcysteine (NAC) remarkably enhanced the HeLa cells’ viability treated with crocetin by 40% via ROS scavenging, whereas the control HeLa cells’ viability did not alter. Crocin co-treatment also elevated the HeLa cells’ viability by 10% which indicates crocin was also able to induce ROS by a smaller amount than crocetin. Hence, high induction of ROS indicates higher anticancer properties for crocetin versus crocin. The observed cytotoxicity of crocin is due to other mechanisms because crocin did not induce ROS significantly (62).

Based on these studies, saffron and its constituents could be considered a chemoprotective agent because of the mechanisms such as activating the Nrf2/HO-1 signaling pathway, NQO1, NQO2, and anti-oxidant enzymes such as SOD and CAT, and inhibiting ROS, NF-κB, and inflammation. 

***Lung protective effects***

In monocrotaline (MCT)-induced pulmonary arterial hypertension (PAH) in Sprague-Dawley rats, the protective effects of crocin (7.5, 15, and 30 mg/kg, intraperitoneal, 21 days) have been studied. An important factor in the PAH pathogenesis is oxidative stress, and oxidation resistance 1 (OXR1) plays a vital role against oxidative stress. MCT-induced PAH via enhancement of lipid peroxidation and reduction of the efficiency of the anti-oxidant defense system that was correlated with reducing the expression of the OXR1 gene and downstream gene targets such as p21 and Nrf2 in lung tissue. Crocin co-treatment revealed a remarkable protective effect on pulmonary arterial pressure in MCT given group in comparison with the PAH alone rats. Besides, crocin-treated groups have shown fewer lung injuries and these beneficial effects of crocin were along with enhanced expression levels of Nrf2, P21, and OXR1 genes. Additionally, the level of GSH biosynthesis and the anti-oxidant enzymes such as CAT and SOD significantly increased in the crocin plus PAH group compared with the PAH group. Thus, crocin treatment could alleviate oxidative stress and lung injuries (63).

Dianat *et al*. (2018) evaluated the effects of crocin (50 mg/kg for 2 months) against cigarette-smoke induced lung injuries and chronic obstructive pulmonary disease (COPD) in Sprague-Dawley rats. HO-1 mRNA expression was significantly reduced in the cigarette smoke (CS) group and crocin co-treatment notably increased mRNA expression of HO-1 which indicates crocin could be a therapeutic option due to its anti-oxidant and anti-inflammatory effects. GSH is a pivotal lung anti-oxidant and its reduction induces alveolar lung damage and airway injury. Glutamate-cysteine ligase catalytic subunit (GCLc) and GSH are regulated by Nrf2. Oxidative stress by the generation of ROS enhanced in the lung tissue through lipid peroxidation which was correlated with reduced expression levels of Nrf2, GCLc, amount of GSH, and other anti-oxidant enzymes. Crocin groups showed lower ROS production resulting in higher GSH and GCLc expression levels. Protein kinase C (PKC), mitogen-activated kinase (MAPK), and phosphatidylinositol 3-kinase (PI3K) provide survival signaling because they are important targets of various anti-oxidant agents. 2-months cigarette smoke exposure reduces the PKC, MAPK, PI3K, and Nrf2 levels but their levels are enhanced by crocin co-treatment. Crocin also attenuated lung injury by reducing the IL-6 and TNF-α levels, leading to decreased inflammation (64).

Bleomycin (BLM) was used in another experiment to induce pulmonary fibrosis in Sprague-Dawley rats, and the lung-protective effects of crocin (20 mg/kg, orally daily for 5 weeks) have been studied. Crocin co-treatment notably reduced the expression of toll-like receptor 4 (TLR4) and IL-10 resulting in its anti-inflammatory effects, decreased TNF-α and transforming growth factor-β1 (TGF-β1) led to its anti-fibrotic effects, enhanced the expression of Nrf2 signaling pathway and HO-1 led to its anti-oxidant effects in the BLM-induced pulmonary fibrosis rats. Thus, crocin could have beneficial therapeutic properties in lung injury (65).

In an *in vitro* study on human lung adenocarcinoma epithelial cells (A549), the crocin (500 μM for 48 hours) protective effects against 1, 2.5, and 5% cigarette smoke extracts (CSE) have been investigated. Crocin administration enhanced the decreased level of Nrf2 and GCL and suppressed ROS resulting in anti-oxidant defense in cytotoxicity induced by CS in A549 cells. Exposure of CS via increasing apoptosis and necrosis induced cytotoxicity in A549 cells. Crocin co-treatment greatly decreased cell injuries. Oxidative stress enhancement is the general pathway in the formation and development of cell injuries. 4-Hydroxynonenal (4-HNE) and MDA enhancement by CS exposure, through the mechanism of the endoplasmic reticulum stress pathway, could induce tissue damage which is decreased by crocin co-treatment. GCL and GSH levels diminished in CS-induced A549 cells but co-treatment with crocin increased their levels. In addition, crocin co-treatment significantly reduced ROS generation and lipid peroxidation (MDA) and increased GSH in comparison with CSE alone. The experiment also revealed the free radicals (O2^·- ^and ^·^OH) scavenging activity and anti-oxidant properties of crocin. Therefore, crocin could be a novel and useful pharmacological approach for pulmonary-related diseases such as COPD (66). 

Taken together, saffron and its constituents could have beneficial properties against lung injury and fibrosis via inducing the Nrf2/HO-1 signaling pathway leading to anti-oxidant effects. Moreover decreasing the inflammation could be considered another mechanism for the lung-protective properties of saffron and its constituents.

***Neuroprotective effects***

To investigate the protective effects of safranal in neurotoxicity induced by rotenone, the expression level of Nrf2 and keap1 in dopaminergic neurons was detected. The results showed that pretreatment with safranal significantly decreased the increased expression level of keap1 and remarkably increased the reduced expression level of the Nrf2 signaling pathway in rotenone-induced neurotoxicity in dopaminergic neurons. These effects indicate that Nrf2 may be associated with the neuroprotective effects of safranal. They also blocked the Keap1/Nrf2 signaling pathway to confirm the results. The knockdown of Nrf2 markedly eliminated the protective effects of safranal against rotenone-induced oxidative stress and apoptosis. These results indicate safranal could have neuroprotective properties by the Keap1/Nrf2 signaling pathway (67).

The effects of safranal (10, 15, 20, and 50 μg/ml for 7 days) against Parkinson’s disease (PD) in rotenone-induced PD in Sprague-Dawley rats have been demonstrated. Safranal administration decreased keap1 expression and induced the Nrf2 signaling pathway and its anti-oxidant genes including GST, GCLc, NQO1, and HO-1 in rotenone-induced neurotoxicity in dopaminergic neurons. Based on these results, safranal administration could protect against neurotoxicity induced by rotenone in dopaminergic neurons through the Nrf2 signaling pathway. Besides, safranal significantly repressed the generation of ROS, oxidative stress, and cell apoptosis in rotenone-induced PD. Hence, safranal could be a therapeutic agent in PD (68). 

Based on these studies, saffron and its constituents could suppress keap1 and activate expression of the Nrf2/HO-1 signaling pathway and related genes such as GST, NQO1, and GCLc leading to decreased oxidative stress. Therefore, saffron and its constituents could have neuroprotective properties.

***Anti-arthritic effects***

A large population worldwide is suffering from the most common form of arthritis, i.e., osteoarthritis. Three important reasons for the progression of this disease are the weakness of muscles, inflammation, and oxidative stress. The possible therapeutic effects of crocin (30 mg/kg) in meniscectomy (MNX) surgery-induced osteoarthritis in rats have been investigated. Crocin administration suppressed the JNK signaling pathway whereas, the ERK signaling pathway was not affected by crocin treatment, IL-6 and NF-κB expression were inhibited by crocin treatment in MNX surgery-induced osteoarthritis in rats. Citrate synthase was also enhanced by crocin treatment. Crocin treatment decreased the increased level of lipid peroxidation and Nrf2 in MNX-induced osteoarthritis in rats. Moreover, crocin administration increased GSH reductase and gamma-GCS. Indeed, GSH level was increased by crocin treatment. According to these results and mechanisms, crocin could be a potential agent in the therapy of osteoarthritis due to its anti-inflammatory and anti-oxidant properties (69). 

In both *in vitro* and *in vivo* experiments, the anti-arthritic impacts of crocetin (12.5, 25, 50, 100, 200, and 400 mg/ml for *in vitro* and 5 mg/kg, 10 mg/kg, and 20 mg/kg for *in vivo* part of the study) have been evaluated. Lipopolysaccharide (LPS) induced mouse macrophages (RAW 264.7) were used for the *in vitro* part and complete Freund’s adjuvant (CFA)-induced arthritis in rats was used for the *in vivo* part of the experiment. Crocetin co-treatment revealed anti-inflammatory effects by suppressing the expression level of iNOS, COX-2, and pro-inflammatory (IL-6, IL-10, IL-1β, and TNF-α) in the *in vitro* part of the study. Nitric oxide also plays a notable role in the induction of inflammation that was decreased by crocetin co-treatment. Furthermore, in a dose-dependent manner, crocetin decreased the increased level of granulocytes and inflammation macrophage migration and increased the reduced level of red blood cells and hemoglobin. NF-κB plays a pivotal role in the degradation of joints and inflammation which was suppressed by crocetin treatment in comparison with the CFA-induced rats. Co-treatment with crocetin also enhanced the expression level of the Nrf2 signaling pathway and HO-1 in comparison with the CFA control groups leading to its anti-oxidant effects. Thus, these results suggested that crocetin could be a therapeutic agent for painful inflammatory and chronic arthritic conditions (70). 

***Anti-colitis effects***

Ulcerative colitis (UC) is correlated with serious systemic complications in the world population. The colon protective and anti-ulcerogenic effects of crocin (20 mg/kg, orally, daily for 8 days) in intracolonic instillation of acetic acid (AA)-induced UC in Sprague-Dawley rats have been studied. Crocin administration markedly reversed AA-induced colonic injury and remarkably attenuated inflammation, oxidative stress, and apoptosis induced by AA in rats. TNF-α and Ca^2+ ^levels were diminished by crocin treatment. Besides, the gene expression level of the Nrf2 signaling pathway and HO-1 were notably enhanced and the activity of caspase 3 was significantly reduced by crocin treatment in AA-induced UC rats. Crocin treatment also increased the level of GSH and decreased the level of lipid peroxidation in AA-induced UC in rats. Thus, the suggested mechanisms of anti-ulcerogenic and colon protective properties of crocin were its anti-oxidant, anti-inflammatory, and anti-apoptotic effects (71). 

In another study, acute colitis was induced by dextran sulfate sodium (DSS) in C57BL/6 mice and the protective effects of saffron (7.5, 15, 20, and 25 mg/kg daily for 8 days) have been investigated. Saffron administration markedly attenuated the histopathological damages in the mucosa of the colon in DSS-induced acute colitis in mice. Pro-inflammatory macrophages were remarkably decreased and anti-inflammatory macrophages were notably increased by saffron treatment in DSS-induced colitis. Moreover, protein expression of HO-1 was significantly enhanced by saffron which indicates saffron protective effects are associated with the Nrf2 signaling pathway. These results and mechanisms reveal the possible potential therapeutic properties of saffron against colitis (72).

***Other therapeutic and biological properties***

Qiu *et al*. (2020), evaluated the hypoglycemic, hypolipidemic, and renal protective properties of crocin (CR) (50 mg/kg, daily for 8 weeks) in old db/db mice. A common mouse model of type-2 diabetes mellitus is db/db mouse. In comparison with effective medications such as metformin, crocin showed better effects on diabetic nephropathy. The fasting blood glucose and glycosylated hemoglobin A1 levels were markedly reduced by crocin. In addition, low levels of insulin and pyruvic kinase were restored by crocin administration. Insulin resistance could lead to hyperinsulinemia, hyperglycemia, and hyperlipidemia. Inadequate insulin levels could result in complications of diabetes. Crocin treatment also elevated SOD, GSH, and CAT led to a decreased level of ROS. Crocin administration recovered the modified level of N-acetyl-beta-D-glucosaminidase and blood urea nitrogen which could be the mechanism for its renal protective effects. The anti-diabetic nephropathy effects of crocin are associated with modulation of NF-κB phosphorylation through activation of the Nrf2 signaling pathway. Thus, crocin could have hypoglycemic, hypolipidemic, and renal protective effects in db/db mice, which are correlated with its anti-inflammatory effects by inducing the Nrf2 signaling pathway (73). Acute pancreatitis (AP) is a disease associated with inflammation and oxidative stress. Cerulein-induced AP was used to investigate the protective effects of crocin (30 mg/kg and 100 mg/kg, daily for 7 days) in male Swiss albino mice. Crocin notably reduced the increased levels of amylase and lipase in comparison with the cerulein group. By crocin administration, the product of lipid peroxidation (MDA) significantly decreased and the decreased level of GSH was restored compared with the cerulein group. Besides, the increased levels of nitrite were diminished by crocin treatment. Surprisingly, in crocin treatment groups, the level of all these mentioned parameters was similar to the normal control group. Moreover, the expression levels of IL-1β, IL-17, TNF-α, and IL-6 were remarkably reduced by crocin administration compared with the cerulein group. Furthermore, crocin treatment decreased the increased level of NF-κB. Nrf2 signaling pathway was also up-regulated by crocin treatment compared with the cerulean group. Taken together, crocin could be considered a potential agent for AP disease due to its anti-inflammatory and anti-oxidant properties (2). In an *in vitro* experiment, the protective effects of crocin on lipopolysaccharide- (LPS-) stimulated RAW 264.7 macrophages have been demonstrated. Crocin induced the protein expression of HO-1 via enhancement of [Ca^2+^]i and subsequent activation of Ca^2+^/calmodulin dependent protein kinase IV, Akt, and Nrf2 signaling pathway in LPS-induced macrophages resulted in its diminishing effect on iNOS expression and NO production in LPS-induced macrophages. NF-κB translocation to the nucleus was suppressed by crocin administration. Cumulatively, crocin could be considered a possible potential supplement for endotoxin-mediated inflammation (74). For inducing preeclampsia-like phenotype, particularly gestational hypertension in Sprague-Dawley rats, N-Nitro-L-arginine methyl ester (L-NAME) was used and the protective effects of crocin (50, 100, and 200 mg/kg/day) administration have been demonstrated. Blood pressure and urine proteins were diminished by crocin treatment in the L-NAME-induced gestational hypertension rats. The experiment also revealed anti-oxidative and anti-inflammatory properties of crocin leading to its protective effects against gestational hypertension in L-NAME-induced preeclampsia-related hypertensive rats. The low activity of the Nrf2/HO-1 signaling pathway is closely correlated with the progression of gestational hypertension. Crocin administration significantly activated the Nrf2/HO-1 signaling pathway which was along with decreasing the symptoms of gestational hypertension (75).

NLRP3 inflammasome is associated with some chronic inflammatory diseases. In an *in vitro* experiment, the effects of safranal (6.25, 12.5, 25, and 50 mg/kg) on mammalian cell line J774A.1 have been evaluated. IL-1β has a pivotal role in the tissue damage of NLRP3 inflammasome. In ATP and LPS primed stimulated J774A.1 cells, safranal treatment, significantly diminished pro-IL-1β processing by regulating CASP1 and CASP8. Safranal pre-treatment markedly enhanced the Nrf2 signaling pathway resulting in its anti-oxidant properties. The anti-NLRP3 effects of safranal were eliminated by Nrf2 knockdown which indicates that its anti-NLRP3 effects are associated with the Nrf2 signaling pathway. Additionally, LPS increased the levels of IL-6 and TNF-α was remarkably reduced by safranal pre-treatment in ATP and LPS primed stimulated J774A.1 cells. NF-κB also was suppressed by safranal pre-treatment. Based on these results and mechanisms, safranal could be considered a preventive agent in NLRP3-related inflammatory diseases (76).

Under physiological conditions, Nrf2 in the cytosol undergoes degradation through ubiquitination and homeostasis. Saffron administration during oxidative stress and inflammation causes Nrf2 induction, its transfer into the nucleus, and protective and therapeutic effects in different organs via its anti-oxidant and anti-inflammation properties

**Table 1:** Protective effects of saffron and its constituents on the different organs mediated by the Nrf2 signaling pathway

**References**	**Results/mechanisms**	**Study design**	**Dosage**	**Constituents**
(72)	↑Anti-colitis effects ↓Nrf2, HO-1, and anti-inflammatory macrophages Pro-inflammatory macrophages	*in* * vivo* /DSS-induced acute colitis C57BL/6	7.5, 15, 20, 25 mg/kg/8 days	Aqueous extract of Saffron
(58)	↓LDH, creatine kinase, oxidative stress, apoptosis, inflammation, Bax, Caspase, MDA, and SOD ↑Nrf2	*in* * vivo* /Isoproterenol-induced myocardial infarction in Wistar rats	50, 100, and 200 mg/kg/ day/15 days	Crocetin
(56)	Heart protective ↓UPR ↑Nrf2, HO-1, NQO1, and NADPH	*ex* * vivo*/IR injury in Sprague-Dawley rats	10 μM, 20 μM, and 40 μM	Crocetin
(70)	↓Arthritic pain, inflammation, oxidative stress, NF-κB, iNOS, COX-2, IL-6, IL-10, IL-1β, and TNF-α ↑Nrf2 and HO-1	*in* * vitro*/ LPS induced arthritis in mouse macrophages (RAW 264.7) And *in vivo* / CFA-induced arthritis in rats	*in* * vitro*: 12.5, 25, 50, 100, 200, and 400 mg/ml/24 hours *in vivo* : 5 mg/kg, 10 mg/kg, and 20 mg/kg/28 days	Crocetin
(62)	↑Cytotoxicity in cancer cells, ROS, oxidative stress, Nrf2, HO-1, NQO1, and NQO2 ↓LDHA, membrane potential, and ATP level	*in* * vitro*/A549, HepG2, HCT-116, HeLa, and SK-OV-3 cancer cell lines	-6 hours hours	Crocetin and crocin
(78)	↓Toxicity, ROS, oxidative stress, mitochondrial membrane potential loss, and Nrf2 translocation	*in* * vitro*/ cyclosporine A-induced toxicity in HEK-293	200, 400, and 600 μM/24 hours	Crocetin and crocetin-loaded lipid nanoparticles
(50)	Hepatoprotective effects ↑SOD, CAT, GPx, and Nrf2 ↓ROS	*in* * vivo* /Liver ischemia-reperfusion injury in male Wistar rats	200 mg/kg/7 days	Crocin
(51)	Hepatoprotective effects ↓Oxidative stress, and ROS ↑Nrf2, HO-1, and NQO1	*in* * vivo* /ATO-induced hepatic injury in rats	25 or 50 mg/kg/7 days	Crocin
(52)	↑Tissue blood flow, rate of survival, hemodynamic parameters, liver function, Nrf2, HO-1, SOD, GSH, and CAT ↓Oxidative stress and ROS	*in* * vivo* /THS-induced hepatic injury Sprague-Dawley rats	60 mg/kg/-	Crocin
(77)	↓Liver injury and oxidative stress ↑Nrf2, SOD, CAT, and GPx	*in* * vivo* /IR-induced hepatic injury in male Wistar rats	200 mg/kg/7 days	Crocin
(54)	↓Cardiac injury, ROS, and NF-κB ↑Nrf2, HO-1, NQO1, Bcl-2, SOD, CAT, and GPx	*in* * vivo* /ATO-induced cardiac injury in male adult Sprague-Dawley rats	40 mg/kg/day and 80 mg/kg/day/7 days	Crocin
(57)	↓Cardioprotective ER stress, GRP78, CHOP, apoptosis, and miR-34a ↑Nrf2, HO-1, and Sirt1	*in* * vivo* /C57BL/6 mice undergoing ER stress and IR injury	50 mg/kg/d for 7 days	Crocin
(59)	↓Malignancy, oxidative stress, inflammation, MDA, and c-JNK ↑TAA, BAX, Bcl-2, TRAIL, p53 expression, Nrf2, HO-1, Keap1, SOD, GPx, GST, and GSH	*in* * vivo* /TAA-induced HCC in Sprague-Dawley rats	200 mg/kg, IP/daily for 4 weeks	Crocin
(60)	↓Development of adenocarcinoma and colitis, oxidative stress, inflammation, NF-*κ*B, COX-2, iNOS, IL-1β, and TNF-α ↑Nrf2 expression	*in* * vivo* /AOM and DSS-induced colonic adenocarcinoma	0, 50, 100, and 200 ppm/15 weeks	Crocin
(63)	↓Lung injury and oxidative stress ↑Nrf2, P21, HO-1, CAT, and SOD	*in* * vivo* / MCT-induced PAH in Sprague-Dawley rats	7.5, 15, and 30 mg/kg, intraperitoneal, 21 days	Crocin
(64)	↓Lung damage, oxidative stress, inflammation, ROS, IL-6, and TNF-α ↑Nrf2, HO-1, PI3K, MAPK, PKC, GCLc, and GSH	*in* * vivo* /Cigarette smoke-induced COPD in Sprague-Dawley rats	50 mg/kg/2 months	Crocin
(65)	↓Inflammation, oxidative stress, fibrosis, TLR4, IL-10, TNF-α, TGF-β1 ↑HO-1, and Nrf2	*in* * vivo* /BLM-induced pulmonary fibrosis in Sprague-Dawley rats	20 mg/kg, orally/5 weeks	Crocin
(71)	Anti-ulcerogenic and colon-protective properties ↓Inflammation, oxidative stress, apoptosis, TNF-α, Ca^2+^, and lipid peroxidation ↑Nrf2, HO-1, and GSH	*in* * vivo* /AA-induced UC in Sprague Dawley rats	20 mg/kg/8 days	Crocin
(73)	↓Hypoglycemic, hypolipidemic, and renal protective effects ↓Fasting blood glucose, hemoglobin A1, ROS, MDA, and NF-κB ↑SOD, GSH, CAT, Nrf2	*in* * vivo* /Old db/db mice	50 mg/kg/8 weeks	Crocin
(2)	Pancreas protective effects ↓Inflammation, oxidative stress, amylase, lipase, MDA, IL-1β, IL-17, TNF-α, IL-6, and NF-κB ↑GSH and Nrf2 signaling pathway	*in* * vivo* /Cerulein-induced AP in male Swiss albino mice	30 mg/kg and 100 mg/kg/7 days	Crocin
(75)	↓Blood pressure, urine proteins, and symptoms of gestational hypertension ↑Nrf2/HO-1 signaling pathway	*in* * vivo* /L-NAME-induced gestational hypertension in rats	50, 100, and 200 mg/kg/day	Crocin
(61)	↓Invasion, migration, malignancy, TAZ, and YAP ↑Nrf2, Bcl-2/Bax ratio, and Caspase	*in* * vitro*/Human gastric mucosa epithelial cell line GES-1 induced by MNNG	0–100 μM/6 weeks	Crocin
(66)	↓Oxidative stress, inflammation, lipid peroxidation, ROS, 4-HNE, and O2^·- ^and ^·^OH ↑Nrf2 and GCL	*in* * vitro*/Cigarette smoke-induced lung cellular toxicity in A549 cells	500 μM/48 hours	Crocin
(74)	↓Inflammation ↑HO-1, [Ca^2+^]i, Ca2+/calmodulin dependent protein kinase IV, Akt, and Nrf2 signaling pathway ↓iNOS and NF-κB	*in* * vitro*/LPS- stimulated RAW 264.7 macrophages	500 𝜇M/24 hours-	Crocin
(67)	↓Neurotoxicity, Keap1, apoptosis, and oxidative stress ↑Nrf2	*in* * vitro*/Rotenone-induced neurotoxicity dopaminergic neurons	-	Safranal
(68)	↓Neurotoxicity, oxidative stress, ROS, and cell apoptosis ↑Nrf2, GST, NQO1, GCLc, and HO-1	*in* * vitro*/Rotenone-induced PD in Sprague-Dawley rats	10, 15, 20, and 50 μg/ml/7 days	Safranal
(76)	Anti-NLRP3 inflammasome properties ↓IL-6, TNF-α, NF-κB, and pro-IL-1β ↑Nrf2 signaling pathway	*in* * vitro*/ATP and LPS primed stimulated J774A.1 cells	6.25, 12.5, 25, 50 mg/kg/0.5 hours	Safranal
(55)	↓Cardiac injury, Inflammation, ROS, MDA, NT, and GSK3b ↑Nrf2, HO-1, NQO1, MnSOD, GSH, NADPH, Akt, and ERK1/2	*in* * vivo* and *in vitro*/IR-induced injury in Wild-Type (WT) and ischemic myocardial tissue	60 µg/kg bolus 15 min and 60 mg/kg/day/4 weeks	Saffron aqueous extract
(53)	↓Liver injury and ROS ↑keap1, Nrf2, HO-1, SOD, GSH, and CAT	*in* * vitro*/Rat liver cell line (BRL-3A)	300 *μ*g/ml/1 day	TFESP, TFESS, and TFEMS

## Conclusion

During oxidative stress and inflammation conditions, the Nrf2/keap1/HO-1 signaling pathway acts against these conditions. One of the natural products that has therapeutic properties through this signaling pathway is saffron. Saffron and its constituents such as crocin, safranal, and crocetin have various pharmacological effects such as tumor suppressor, anti-inflammatory, anticarcinogenic, anti-oxidant, and antigenotoxic actions. Saffron and its constituents could be therapeutic and protective agents in different pathological conditions in different tissues such as the liver, heart, pancreas, lungs, and brain through the Nrf2 signaling pathway. Moreover, multiple studies have shown that saffron and its constituents by affecting the Nrf2/HO-1 signaling pathway can prevent some diseases such as cancer, ulcerative colitis, and diabetes by different mechanisms such as inducing anti-oxidant enzymes (SOD, CAT, and GPx), reducing lipid peroxidation (MDA) and inflammation, inducing apoptosis in cancer cells and particularly inducing the Nrf2/HO-1 signaling pathway leading to reducing oxidative stress and inflammation.

## Authors’ Contributions

AK Preparation of original draft; BMR Critical revision of the paper, supervision of the research; HH Study conception, design and supervision of the research. All authors have agreed to the contents and approved the final version for publication 

## Conflicts of Interest

The authors have no conflicts of interest to declare.
